# Classification of Pharmaceutical Policy Measures During the Portuguese Financial Crisis

**DOI:** 10.1177/00469580221093171

**Published:** 2022-05-19

**Authors:** António Augusto Donato, João Rui Pita, Francisco Batel-Marques

**Affiliations:** 1 School of Pharmacy, Laboratory of Social Pharmacy and Public Health, University of Coimbra Faculty of Pharmacy, Coimbra, Portugal

**Keywords:** pharmaceutical policy, economic recession, pricing, reimbursement, prescription, generics

## Abstract

**Background:**

A rise in pharmaceutical expenses in Portugal led to the introduction of policy measures aimed at controlling outpatient public costs. This research examines and categorizes the most common pharmaceutical measures implemented during the Troika intervention, as well as comparing this period of time to prior ones.

**Methods:**

A hierarchical structure of descriptors was built to classify and group measures over a 20-year period, including whether they might be deemed austerity measures. The nature, relative weight, and frequency of measures, along with the evolution of public drug expenditure, were assessed.

**Results:**

Although there were fluctuations, frequency tended to increase. The highest number of policy changes per year was in 2010, a year before the financial assistance. The Troika intervention was characterized by a strong emphasis on pricing and prescription-related initiatives. Generic medicines played a significant role in the effort to reduce public drug expenditure.

**Conclusions:**

During the Troika intervention, outpatient public drug expenditure was consistently reduced through a comprehensive “package of measures” aimed at both the demand and supply sides. The effectiveness of some previous independent measures, if any, was temporary.

**Highlights**

**What do we already know about this topic?**

- In Portugal, austerity measures, particularly direct price cut aimed at reducing outpatient pharmaceutical expenditure, did not appear to have a long-term impact.

**How does your research contribute to the field?**

- A hierarchical structure for organizing and classifying pharmaceutical reforms over time may be a useful tool for identifying distinct policy possibilities for controlling pharmaceutical expenditure.

**What are your research’s implications toward theory, practice, or policy?**

- The study suggests that indirect pricing strategies, such as “External Price Referencing,” and moving toward a more “rational use of medicines” through prescription monitoring and facilitating generic entry and competition, may lead to more sustainable expenditure management.

## Introduction

The 2008 global crisis along with the Portuguese high external debt led to a financial bailout and external financial intervention.^1^ A Memorandum of Understanding (MoU) was signed in May 2011 between the Portuguese Government and the European Commission (EC), the European Central Bank (ECB) and the International Monetary Fund (IMF), collectively known as Troika.^2^ Amongst others, the MoU included quantitative public pharmaceutical expenditure targets expressed as a % of GDP, but no split was made between hospital and ambulatory care: 1.25% of GDP by the end of 2012 and around 1% of GDP in 2013 (in line with the EU average)”.^2^

Before Troika, the measures Portugal used to contain pharmaceutical expenditure proved to be ineffective.^3,4^ During Troika’s intervention (2011-2014), Portuguese National Health System (PHNS) expenditure in the outpatient sector decreased from .91% of GDP in 2010 to .70% in 2012, and to .68% in 2013.^5-7^ Total PHNS pharmaceutical expenditure (hospital and outpatient) decreased from 1.47% in 2010 to 1.30% in 2012, and to 1.23% in 2013.^5-7^

Countries define pharmaceutical policies to balance access to medicines with public expenditure. Prior to Troika assistance (2009–2011), there was a decrease in annual medicines utilization; however, during the intervention, there was a sustained reduction in pharmaceutical expenditure that did not jeopardize medicines utilization.^4^

The aim of this study was to investigate the type, number, and mix of policy measures employed during Troika’s financial rescue. Three aspects were studied: (a) whether or not the policy measures differed from those previously utilized, (b) if they aligned to the priorities defined in the MoU, and (c) evolution of measures in relation to outpatient pharmaceutical expenditure.

### Descriptors

To categorize pharmaceutical policy measures, a hierarchical classification of descriptors was used. A descriptor was defined as a word (or two words) used to identify and describe different types of pharmaceutical policy measures. The descriptors from the MoU were: “Pricing,” “Reimbursement,” “Prescription and monitoring of prescription” and “Pharmacies sector”.^2^ The hierarchical classification resulting from the MoU was compared to frameworks that were used to classify and evaluate the impact of policy measures during the same period of time.^8-13^ The MoU was able to reproduce the most commonly used descriptors, with the exception of the individualization of “Generic policies.” Though not considered as an individual category, the majority of the specific measures included in the MoU focused on such policies. To improve reproducibility, “Generic policies” was included in our hierarchical classification. We also introduced “Others” to cover less common measures. After a final revision according to the PPRI Multi-language Glossary of Pharmaceutical Terms,^14^ the first level of descriptors was set as follows: “Pricing,” “Reimbursement,” “Prescription,” “Community Pharmacy,” “Generic policies,” and “Others.”

Based on published literature^8,10-17^ the first six descriptors were further divided into a second level, including whether or not they could be classified as austerity measures.

Cost-containment policies, that is, austerity measures, usually comprise tax increases, expenditure cuts, or a combination of both.^18^ Similar to Vogler et al^8^ austerity measures were classified as policy adjustments focused on lowering public pharmaceutical expenditure in the short term. Other measures, such as those aimed at increasing generic use or contributing to more responsible prescription and use of medicines, might have led to savings but were not considered austerity measures.

We did not conduct a systemic review. The legislation database was retrieved and compiled from online legislation resources, first from the National Authority of Medicines and Health Products (INFARMED),^19^ then compared to the databases of the Portuguese Pharmaceutical Industry Association (APIFARMA)^20^ and of the Portuguese Association of Generics and Biosimilar Medicines (APOGEN).^21^ Finally, data was cross-checked against the online Official Journal of Portuguese Legislation.^22^

### Weight of Descriptors

For a given period of time, the weight is the number of descriptor measures divided by the total number of measures, expressed as a percentage. It was calculated for the global period under analysis and for the periods before and during the Troika intervention. It permitted the comparison of differences in mix of measures before and during the intervention.

### Frequency

The following definitions were established:

Frequency = absolute number of new measures implemented each year, per descriptor;
Sum of Frequencies = total number of measures taken throughout the 20-year period, per descriptor;
Frequency Index (FI):

$$FI\left(year\;X\right)=\frac{frequency\left(year\;X\right)}{sum\;of\;Frequencies}\left(\%\right)$$

Total Frequency = sum of the absolute number of new measures implemented each year; To study the evolution of each descriptor over the period under analysis, FI cumulative percentages were plotted on the *y*-axis of a graphical representation.

## Results

### Hierarchical Structure of Descriptors

The hierarchical structure developed to group and classify pharmaceutical policy measures is presented in Table 1. The full list of surveyed measures assigned according to the descriptors is available in Supplemental Annex 1.

**Table 1.** Descriptors.

Descriptors		Definition	Austerity (Y/N)
Pricing	Price set	Price regimes for different types of medicines: Outpatient market and hospital market; prescription and non-prescription products; branded and generic medicines; entity responsible for price mechanism regulation	No
	External price referencing (EPR)	The practice of using the price(s) of a medicine in 1 or several countries in order to derive a benchmark or reference price for the purposes of setting or negotiating the price of the product in a given country. Reference countries are normally chosen based on a list of European Union countries with a GDP per capita equivalent in purchasing power parity or other periodically defined criteria. The selected countries serve as a reference both for the setting of new maximum prices and for the annual price review	Yes, if countries are selected due to their lower prices, to generate rapid savings, and only price reductions can be admitted
	Parallel trade	A form of arbitrage, within the European Economic Area (EEA), in which medicines are purchased in 1 country, typically where income levels are relatively low, and sold into other countries, where income levels and hence prices are higher	No, free movement of goods aims to improve competition for the consumers’ benefit
	Price cut	A cost-containment measure during which the set price of a medicine is reduced by the authorities	Yes
	Price review	Evaluation of the price of all, or groups of, medicines, typically in comparison to the prices of the same medicines in other countries, in order to account for developments such as the market entry of medicines and price changes in other countries and exchange rate evolutions. Price reviews may, or may not, be performed in combination with reimbursement reviews. Price reviews can be done systematically (e.g., once a year) or out-of-schedule	Yes, if criteria are defined to generate rapid savings due to budgetary constraints
Reimbursement	Health technology assessment (HTA)	For non-generic drugs subject to medical prescription, it is a multidisciplinary process that summarizes information about the medical, social, economic and ethical issues related to the use of a health technology in a systematic, transparent, unbiased, robust manner, for decision making on reimbursement, and in Portugal, determines the launching price of a reimbursed medicine	No, it is an analysis of alternatives according to their cost-effectiveness
	Reimbursement list	A list that contains medicines with regard to their reimbursement status. It may either include medicines eligible for reimbursement (positive list) or those explicitly excluded from reimbursement (negative list). Reimbursement lists may target either the outpatient sector (usually positive lists or negative lists) or the in-patient sector (typically called hospital pharmaceutical formulary), or both. Cf. Positive list, negative list	No, unless it is a transfer of costs from the payer to the patient to achieve quick savings due to budgetary constraints
	De-listing (delisting)	Exclusion of a medicine from a reimbursement list (e.g., positive list), often resulting in exclusion from reimbursement	No, as reimbursement reviews are a valid monitoring mechanism aimed at increasing efficiency
	Reimbursement rate	The percentage share of the price of a medicine or medical service that is reimbursed/subsidized by a public payer. The difference between the reimbursed amount and the full price of the medicine or medicinal service is paid by the patient	Yes, if it is a decrease in the rate
	Eligibility Scheme(s)	Population group-specific reimbursement For certain population groups (e.g., children, the socially disadvantaged), separate schemes apply in which medicines are reimbursed in full or at a higher rate	No, unless it is a transfer of costs from the payer to the patient to achieve quick savings due to budgetary constraints
	Reference price system (RPS)	A reimbursement policy in which identical medicines (ATC level 5) are clustered (reference group). The public payer funds a maximum amount (the reference price), while the patient must pay the difference between the reference price and the actual pharmacy retail price of the medicine, in addition to any co-payments (such as prescription fees or percentage co-payment rates)	No, because it is a policy aimed at increasing efficiency through the competitive use of medicines, especially generics
Generic policies	Generics pricing	Price link: Practice of setting the price of a generic in relationship to the originator medicine, usually at a certain percentage lower than the originator medicine price. The design of this generic price link policy may vary, with different percentages for the different generics (the first generic coming to the market, second generic, etc.), and in some cases the prices of originator medicines might also be part of the policy, that is, that they will also be required to decrease Price cut: A cost-containment measure during which the set price of a generic medicine is reduced by the authorities	No, unless it aims to reduce or cut generic prices
	Generics regulation	Policies, regulations, measures and initiatives that promote the use of generic drugs, usually carried out by government authorities	No, given that these demand-side measures are aimed at encouraging increased generic uptake
	Patients’ incentives	Patient information on medication prices and how they can save money with generics	No, given that these demand-side measures are aimed at encouraging increased generic uptake
	Physicians’ incentives	Fixed budgets applicable to primary care physicians provide an explicit incentive to contain costs, which encourages the prescription of generics. Incentives may be set up to reward doctors who spend less, punish doctors who spend too much, or do both	No, given that these demand-side measures are aimed at encouraging increased generic uptake
	Pharmacists’ incentives	Pharmacists are compensated in order not to discourage them from dispensing the less expensive product	No, given that these demand-side measures are aimed at encouraging increased generic uptake
	Breakdown artificial barriers for generic market access (e.g., patent linkage)	Originators put pressure on regulatory authorities using strategies such as sending warning letters accusing them of patent infringement, or initiating administrative procedures to revoke marketing authorizations, price and reimbursement of generics	No, given that these measures are aimed at facilitating generics entry
	International non-proprietary name prescribing (INN prescribing)	Requirements for prescribers (e.g., physicians) to prescribe medicines by its INN, that is, the active ingredient name instead of the brand name	No, given that these demand-side measures are aimed at encouraging increased generic uptake
	Generic substitution	The practice of substituting a medicine, whether marketed under a trade name or generic name (branded or unbranded generic), with a less expensive medicine (e.g., branded or unbranded generic), often containing the same active ingredient(s)	No
Prescription	Pharmaceutical promotion and interaction with health care professionals (HCPs)	All kinds of information and promotional activities to doctors that provide incentives with the aim of influencing prescription of pharmaceuticals. Measures to encourage transparency and to control interaction between the pharmaceutical industry and HCPs	No
	Electronic prescription	Fast and efficient mode of prescription, enables guidance and forms to be introduced, encourages correct and up-to-date awareness of generic drugs, prices, enables auditing, control and combating fraud	No
	Prescription forms and information systems	To increase efficiency, cost containment, enables auditing, control and combating fraud	No
	Prescription guidelines	Instructions to physicians to ensure rational prescribing of medicines (i.e., to ensure that the right medicine in the right dose is given to the right patient at the right time)	No
Community pharmacy	Ownership, licensing, establishment and operation	Limiting, or not, ownership to pharmacists and defining the number of pharmacies per owner. License based on demographic and geographical criteria provided by the national authority needed to open and operate a community pharmacy in a specific location, including opening hours, workforce, premises, equipment, and existence of responsible pharmacist, and regulation of the pharmacy workforce	No
	Discounts, rebates, loyalty schemes	Generic manufacturers may offer discounts, rebates or promotions to pharmacies in order to gain an advantage over their competitors	No
	Distribution remuneration	Refers to payments for the services of wholesalers and pharmacies. The remuneration can be through a fixed percentage on the final retail price, regressive margin schemes or, only for pharmacies, by the payment of a “fee for service.”	Yes, if changes imply decreases in remuneration and savings for public expenditure
	Forms of dispensing medicines	Switch: Reclassification of a prescription-only medicine (POM) to a non-prescription medicine (NPM)/Over-the-counter (OTC) medicine	No
		Dispensing OTC medicines outside community pharmacies	No
		Internet pharmacy (online pharmacy)	No
		Unit dose system	No
Others	Changes in the value-added tax (VAT) on medicines	Decreases or increases in the VAT on pharmaceutical products	Yes, in the case of an increase in the VAT rate on medicines
	Claw-back (and other measures applied in the case of excess spending in pharmaceutical budget)	A funding element in a reimbursement system allowing third-party payers to recoup (part of the) discounts/rebates granted by various stakeholders, such as wholesalers and pharmacists	Yes, if characterized as a reduction/control in public pharmaceutical expenditure because the pharmaceutical industry, wholesalers and pharmacists absorb the potential growth
	Price labeling	Certain forms of labeling of the medicinal product, such as the price, may be required	No

### Total Frequency and Weight of Descriptors

**Figure 1.**
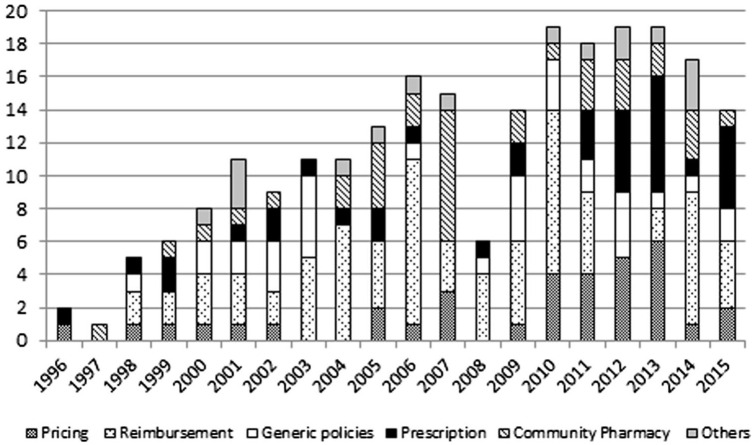
Total frequency.

During the overall period, 234 measures that matched the descriptors’ criteria were found. From 1996, although there were fluctuations, frequency tended to increase. Due to the very low number of measures observed, 2008 was an outlier. The peak of frequency was reached in 2010, a year before the Troika intervention. During the intervention it remained high until 2013, when it began to fall (Figure 1).

**Figure 2.**
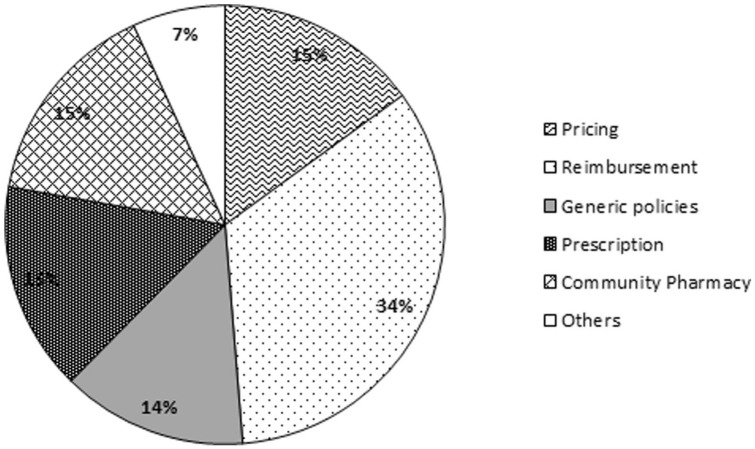
Relative weight of descriptors (20-year period).

Measures aimed at “Reimbursement” were the most employed policies (34%) during the 20-year period. Interventions related to “Pricing,” “Prescription,” and “Community Pharmacy” accounted for 15% each and “Generic policies” for 14% (Figure 2).

**Figure 3.**
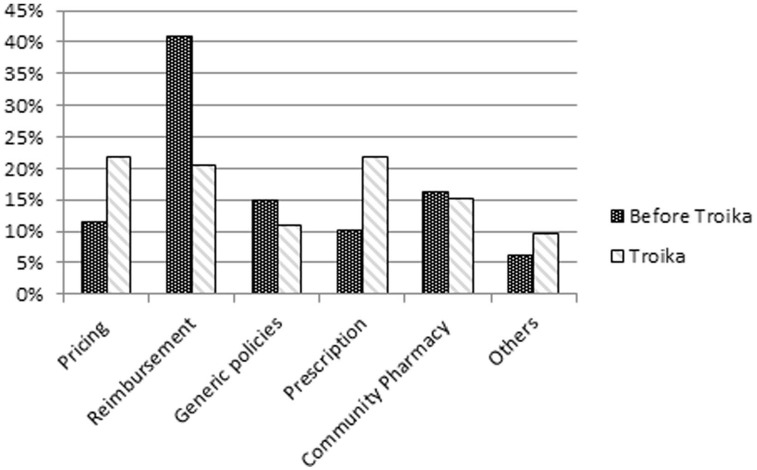
Relative weight of descriptors before and during the Troika intervention.

When comparing the period of time before and during the financial rescue (2011–2014), a shift could be observed: greater emphasis on measures related to “Pricing” and “Prescription” (Figure 3).

### Frequency Index per Descriptor

**Figure 4.**
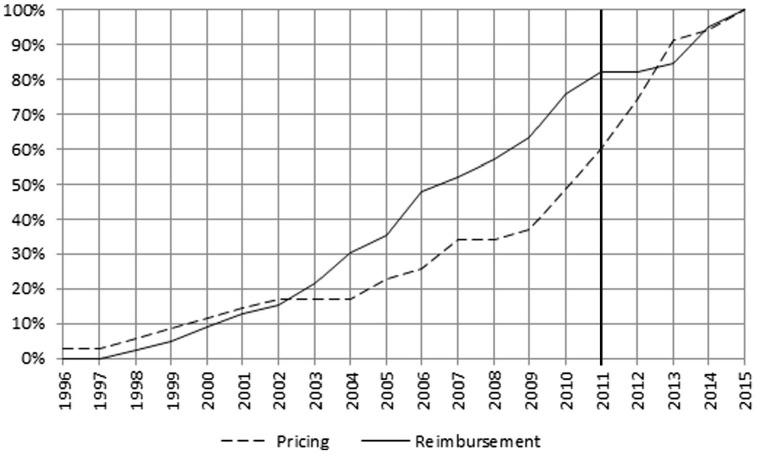
Cumulative percentages FI—Pricing and Reimbursement.

Measures impacting “Reimbursement” were adopted uniformly over nearly the entire 20-year period, but showed a decline during the first 2 years of the Troika intervention (Figure 4).

**Figure 5.**
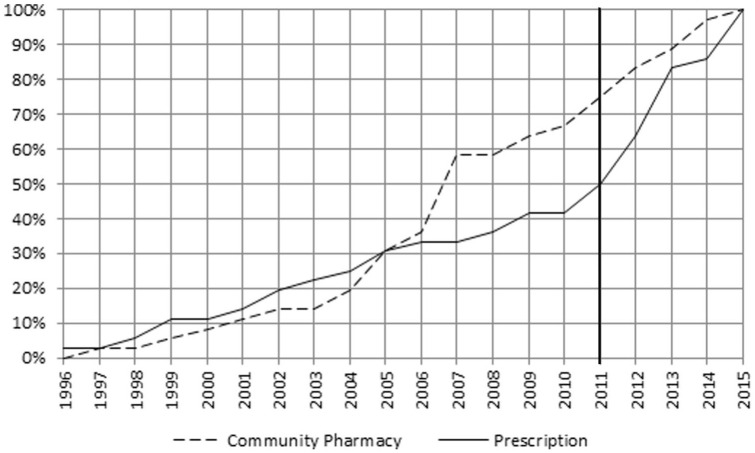
Cumulative percentages FI—Community Pharmacy and Prescription.

**Figure 6.**
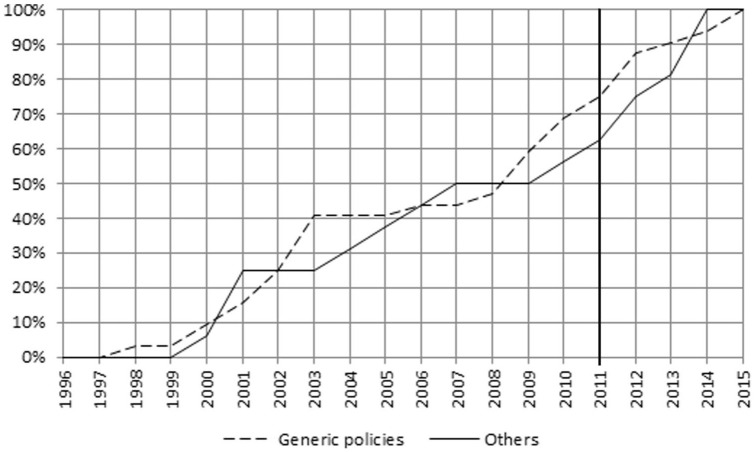
Cumulative percentages FI—Generic policies and Others.

**Figure 7.**
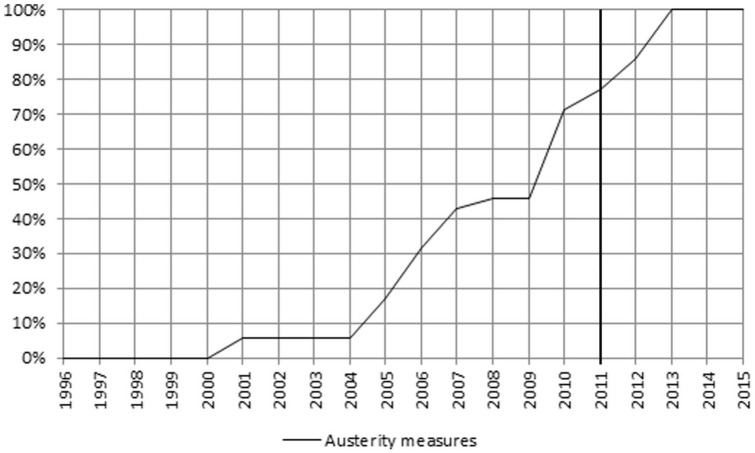
Cumulative percentages FI—Austerity measures.

**Figure 8.**
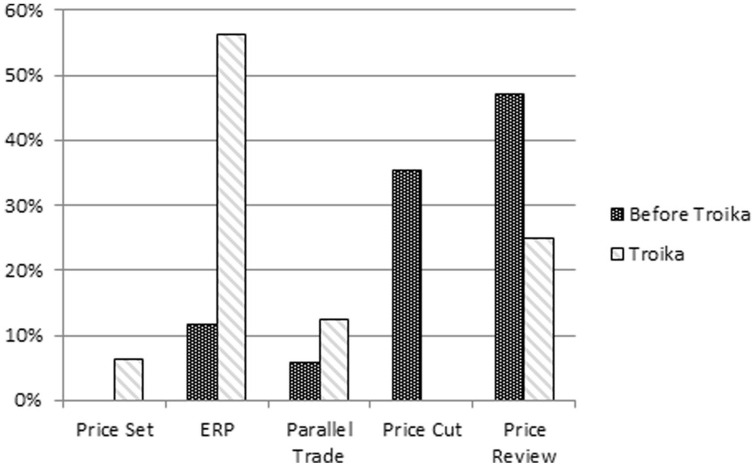
Pricing—relative weight of sub-descriptors before and during the Troika intervention.

The frequency of other descriptors changed over time as depicted in Figures 4 to 8. The main focus of the intervention phase was on “Pricing” and “Prescription.” The cumulative FI of the descriptors between 2011 and 2014 was “Pricing” 46%, “Prescription” 44%, “Community Pharmacy” 31%, “Generic policies” 25%, and “Reimbursement” 19%.

Legislative initiatives in the field of “Generic policies” and “Community Pharmacy” were particularly concentrated during the years 2000–2003 and 2004–2007, respectively.

The intensity of austerity measures was higher during two time periods, 2005–2007 and 2010–2013. The latter accounted for 58% of the total austerity measures, more than half of them aimed at “Pricing.” During 2014 and 2015, no austerity policies were observed (Figure 7).

### Analysis per Sub-descriptor

In addition to the quantitative analysis, the evaluation of the sub-descriptors and individual measures allowed a possible conclusion as to their impact.

**Figure 9.**
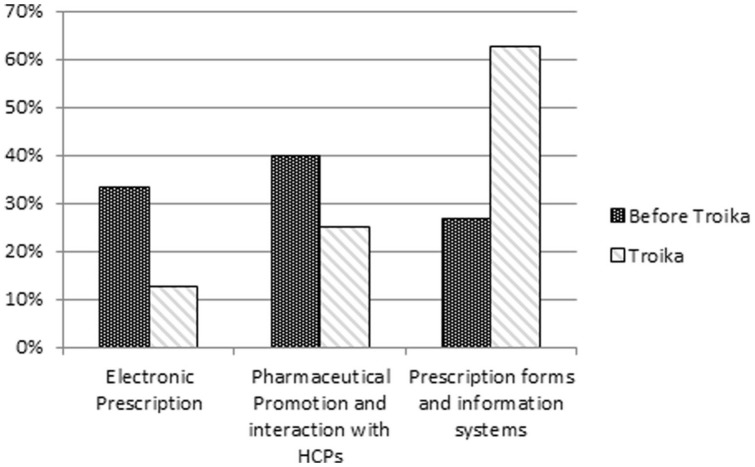
Prescription—relative weight of sub-descriptors before and during the Troika intervention.

The points to highlight during the intervention were the following:

a)“Pricing”—focus changed from the previous administrative price reductions, “Price Cuts,” to an indirect control mechanism through “External Price Referencing” (Figure 8).
b)“Prescription”—most common measures were related to “Prescription forms and information systems” (Figure 9); conformity with the MoU objectives by the dematerialization of prescriptions^1^—“Electronic Prescription”—and by the mandatory disclosure of pharmaceutical industry promotional efforts and interactions with health professionals on the INFARMED website—“Pharmaceutical Promotion and interaction with Health Care Professionals” (Supplemental Annex 1).
c)“Generic Policies” (and using the “austerity” filter)—the same trend as for “Pricing” was observed; from a direct “Price Cut” of 30% in the generics price in 2008, to measures based on “Generic Price Link” increasing differences between originator and generic prices during the intervention (Supplemental Annex 1).

### Pharmaceutical Expenditure

**Figure 10.**
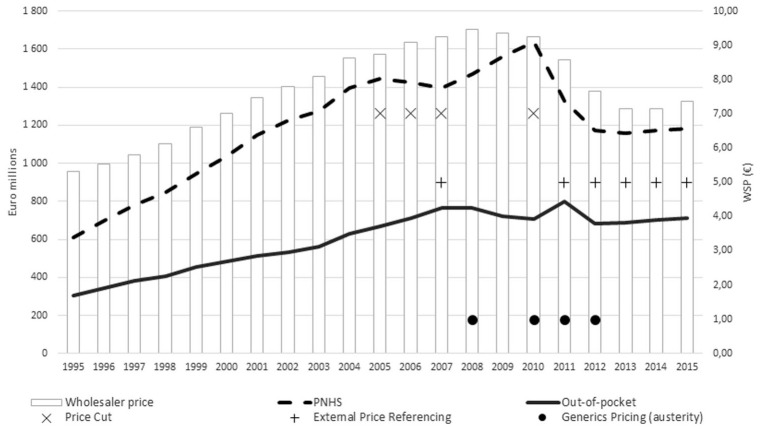
Outpatient pharmaceutical expenditure (PHNS and out-of-pocket) and average wholesale price.

Figure 10 shows the evolution of outpatient pharmaceutical expenditure (both PHNS and out-of-pocket) and wholesaler price, while also highlighting changes in the sub-descriptors “Pricing” and “Generic policies."

Prior to the Troika intervention, public pharmaceutical expenditure only declined throughout the direct price-cutting phase (2005–2007), but then started to rise again.

During the Troika rescue phase, PNHS expenditure decreased and then stabilized. Out-of-pocket spending peaked in 2011, then fell and stabilized. The average wholesale price declined. These changes occurred concurrently with the implementation of “External Price Referencing” and “Generic Price Link” policies.

## Discussion

The present work provided an overview of pharmaceutical policy measures along with the evolution of public drug expenditure in the outpatient sector, during a 20-year period. During this period, the Portuguese economy went through three recessions, 2002–2003, 2008–2009, and 2010–2013.^23^

Previous research pointed out that between 2010 and 2016, Portugal implemented a high number of policy measures related to pharmaceuticals.^8^ This study supported these findings. Between 2010 and 2013, a high frequency of measures was observed, including austerity measures.

The MoU was signed in May 2011,^2^ and the type, frequency, and combination of measures implemented, as well as the trajectory of outpatient pharmaceutical expenditure, differed from those observed during the previous periods of the study.

Two decreasing phases of public expenditure as a percentage of GDP were observed: 2006–2007, and 2011 onwards (Figure 10). Both periods were marked by the deployment of austerity policies.

The first time-segment was characterized by two generalized administrative price cuts as well as changes in the regulations for annual price reviews.^3,16^ This direct price regulation had a temporary effect and resulted from the shift of the financial burden to the patients.^3^

During the second period, which encompasses the Troika intervention, the most frequent measures were “Pricing” related, these having already started in 2010, and being predominant among the policies classified as austerity. Contrary to what was observed before, “Pricing” measures shifted from direct “Price Cuts” to a greater emphasis on price control by “External Price Referencing.” The reference countries were altered, and purchasing power parity and lower price level were used as comparison criteria, as stipulated in the MoU.^2^ There was a prolonged decrease in public pharmaceutical expenditure that was not only explained by a shift to patients. A disaggregated analysis also suggested a transfer of the burden to the patient side between 2010 and 2011, but the expenditure for both the public and patients fell after 2011.^4,5^

**Table 2.** Outpatient pharmaceutical expenditure as a % of GDP.

*Year*	*Gross domestic product at market prices (current prices, million euro, annual)*	Outpatient pharmaceutical expenditure as a % of GDP		
		PNHS	Out-of-pocket	Total
2010	179 929.8	0.91%	0.39%	1.30%
2011	176 166.6	0.75%	0.45%	1.21%
2012	168 398.0	0.70%	0.41%	1.10%
2013	170 269.3	0.68%	0.40%	1.09%
2014	173 079.1	0.68%	0.41%	1.08%
2015	179 809.1	0.66%	0.39%	1.05%

*Source*. PORDATA, 2019; INFARMED 2019.

“Pricing” measures were the most frequently cited measures in countries hit by the 2010–2015 financial crises,^8^ and this study verifies this fact.

Measures affecting “Reimbursement” were the most frequently employed over the whole 20-year period, but this research shows that they might not have been a priority during the Troika intervention, particularly during the first 2 years of implementing the MoU.

European experience suggests that there is no single approach to developing generic policies in Europe.^24^ The Troika intervention was not distinguished by a high frequency of measures aimed at “Generic Policies,” but more by a focus on two priorities, both defined in the MoU: lowering the price of generic medicines, and supporting their development, either by removing barriers or facilitating their prescription and substitution.^2^ The price of generics was reduced by raising the price differential with the originator drug—“Generic Price Link”—and mandating an annual review of generic prices. Compulsory electronic prescription in favor of INN was adopted in both the public and private sectors, and a new system for resolving industrial property problems and overcoming administrative/legal barriers to generic entry was defined (Supplemental Annex 1). From 2010 to 2014, a −53% decrease in the average price of generic drugs was observed.^6^ During the same time frame, the market share of generics, in standard units, increased from 31.4% to 46.5%.^6,7^

The simultaneous use of demand-side incentives and volume controls was found to be necessary to contain pharmaceutical expenditure.^25^ The Troika intervention period was characterized by a high concentration of “Prescription” oriented interventions. The MoU highlighted policies aimed at enhancing “the monitoring system of prescription of medicines.” Initiatives were launched to improve transparency and control interaction between the pharmaceutical industry and HCPs, to enable auditing, control, and combat fraud ( Supplemental Annex 1).

Updates to the regulatory framework for community pharmacies were adopted during the Troika intervention period. The profit margin calculation for pharmacies and distributors was altered to a regressive mark-up and a flat fee. The MoU defined that if the new profit margin method did not result in the anticipated savings, a pay-back would be calculated on the mark-up. Initiatives to allow third-party payers to recover part of the discounts given to pharmacies by generic manufacturers and wholesalers were explored in European nations^26,27^ but were not observed in Portugal during the study period.

It was uncertain if government-industry agreements were effective in expenditure-controlling.^28^ Nonetheless, the Troika intervention period saw annual claw-back agreements between the pharmaceutical industry and the government, imposing expenditure limits. It should be highlighted that no more austerity measures were introduced between 2013 and 2015, indicating that these mechanisms may have been successful.

## Limitations

There were some limitations to the current research. Several initiatives were implemented concurrently throughout the Troika intervention, and only their cumulative influence on pharmaceutical expenditure could be examined, rather than the impact of each 1 individually. Other variables, such as the impact of patent expiration on expenditure containment or the rate of introduction of more expensive innovation in the outpatient sector, could confound our analysis. Pharmaceutical policies interact with 1 another, and their impact tends to diminish over time, but our study only extended to a year after the intervention ended.

## Conclusions and Future Implications

The nature of the measures observed in this study was not different to that described in the literature, but over time there appear to have been shifting priorities in terms of the nature, frequency and combination of the measures. To the best of our knowledge, this was the first paper to categorize and examine the implementation of measures used to regulate the outpatient pharmaceutical sector in Portugal over such a long period of time.

Previously, austerity measures aimed at controlling outpatient drug expenditure appeared to have a temporary impact. The measures implemented during the Troika intervention were in accordance with the memorandum. Beginning in 2011, outpatient public pharmaceutical expenditure fell steadily, coinciding with the implementation of the MoU’s measures.

On the supply side, the most common policies were “Pricing” oriented, while on the demand side, they were “Prescription” oriented. A significant increase in prescription monitoring and control mechanisms was observed, which had not previously been the case.

“Pricing” measures were not focused on administrative and direct price cuts, which were proven to be ineffective; a more sustained control may have been achieved by changing the “External Price Referencing” system and changing the percentage of a generic’s price in relation to the originator medicine (“Generic Price Link”), in parallel with an increase in the share of generic medicines.

Moves were made in the direction of a more “rational use of medicines,” by means of monitoring of prescription (although the control mechanisms were focused on expenditure and quantity of prescriptions) and facilitating generic entries, and enhancing competition between substitute therapies.

Pharmaceutical expenditure projections are typically short-term and based on prior expenditure trends.^29^ After the Troika intervention, we did not identify structured mid-term plans. A horizon-scanning system that makes use of data on expenditure, measures and access should be evaluated in order to help policy makers.

## Supplemental Material

sj-docx-1-inq-10.1177_00469580221093171 – Supplemental material for Classification of Pharmaceutical Policy Measures During the Portuguese Financial Crisis Click here for additional data file. Supplemental material, sj-docx-1-inq-10.1177_00469580221093171 for Classification of Pharmaceutical Policy Measures During the Portuguese Financial Crisis by António Augusto Donato, João Rui Rui Pita and Francisco Batel-Marques in INQUIRY: The Journal of Health Care Organization, Provision, and Financing

## Notes

1. Implemented through a sequential process that was completed in 2015, after the program’s completion, but with the country still subject to regular post-program supervision
