# Supplementary material for: Classification of Pharmaceutical Policy Measures During the Portuguese Financial Crisis
Source: Inquiry. 2022 May 19;59:00469580221093171. doi: 10.1177/00469580221093171 (PMC9127866; doi:10.1177/00469580221093171)
Supplement: sj-docx-1-inq-10.1177_00469580221093171 – Supplemental material for Classification of Pharmaceutical Policy Measures During the Portuguese Financial Crisis [file sj-docx-1-inq-10.1177_00469580221093171.docx]

| Date | Legislation | Content | Descriptor | Sub-descriptor | Austerity measure |
| --- | --- | --- | --- | --- | --- |
| 15/02/1996 | Normative Dispatch no. 8/96 | Price revision of non-reimbursable medicines | Pricing | Price Review | No |
| 17/04/1996 | Order no. 123/96 | Regulation of the National Council for the Advertising of Medicines | Prescription | Pharmaceutical Promotion and interaction with Health Care Professionals (HCPs) | No |
| 07/08/1997 | Resolution no. 220/97 | New license model for retail pharmacies | Community Pharmacy | Ownership, licensing, establishment and operation | No |
| 26/02/1998 | Normative Dispatch no. 22/98 | Definition of the coefficient to be applied in the price reviews of non-reimbursable medicines | Pricing | Price Review | No |
| 08/06/1998 | Order no. 981/98 | Approval of the models of medical and veterinary prescription forms, registration and requisition of narcotic drugs and psychotropic substances | Prescription | Prescription forms and information systems | No |
| 17/09/1998 | Decree-Law no. 291/98 | Update of legal definitions and classification of generic drugs, aiming to increase use, manufacture, distribution and prescription | Generic policies | Generics Regulation | No |
| 28/09/1998 | Dispatch no. 17990/98 | Definition of reimbursement of glucose test strips | Reimbursement | Reimbursement List | No |
| 07/10/1998 | Decree-Law no. 305/98 | Exclusion criteria for reimbursement, reduced proven therapeutic efficacy, excessive cost, adequacy of package size, restriction to certain therapeutic indications; reimbursement items | Reimbursement | De-listing (Delisting) | No |
| 29/01/1999 | Order no. 73/99 | Reference index for the price reviews of reimbursable medicines | Pricing | Price Review | No |
| 16/02/1999 | Decree-Law no. 48/99 | Adaptation to the spirit Council Directive 92/28/EEC of 31 March 1992 concerning the relationship between the pharmaceutical industry and persons qualified to prescribe and dispense medicinal products | Prescription | Pharmaceutical Promotion and interaction with Health Care Professionals (HCPs) | No |
| 26/05/1999 | Dispatch no. 13621/99 | Access to the medicine Aricept® for the treatment of Alzheimer's | Reimbursement | Reimbursement List | No |
| 29/09/1999 | Order no. 1193/99 | Changes to the special prescription form models | Prescription | Prescription forms and information systems | No |
| 22/10/1999 | Order no. 936-A/99 | Rules and conditions for opening and transfer of pharmacies (capitation, distance) | Community Pharmacy | Ownership, licensing, establishment and operation | No |
| 30/10/1999 | Order no. 982/99 | Reimbursement levels for pharmacotherapeutic groups and subgroups; reimbursement increased for neuroleptic and antidepressant drugs when prescribed by psychiatrists or neurologists | Reimbursement | Reimbursement List | No |
| 24/03/2000 | Dispatch no. 6100/2000 | Access to the medicines Exelon® and Prometax® for the treatment of Alzheimer's | Reimbursement | Reimbursement List | No |
| 08/08/2000 | Law no. 14/2000 | Measures to rationalize the medicine policy and increase the use of generic medicines; prescription by INN when there are authorized generic drugs | Generic policies | International Non-proprietary Name Prescribing (INN Prescribing) | No |
| 01/09/2000 | Decree-Law no. 205/2000 | Reimbursement levels, namely D; 15% increase for special regime (poor and pensioners); 10% increase for Generics, reimbursement items; exclusion from reimbursement; systematic reassessment | Reimbursement | Eligibility Scheme(s) | No |
| 05/09/2000 | Order no. 713/2000 | Definition of free pricing for non-reimbursed OTCs, maximum channel margins and supervision by the regulator | Pricing | Price Review | No |
| 26/09/2000 | Decree-Law no. 242/2000 | Definition of Generic Medicine; measures to facilitate the switch to generic drugs; redefinition the forms of identification, prescription and dispensation | Generic policies | Generics Regulation | No |
| 28/09/2000 | Dispatch no. 22651/2000 | Scientific evidence of efficacy and effectiveness for the therapeutic indications, associated with the benefit-risk assessment for inclusion and exclusion from reimbursement | Reimbursement | Reimbursement List | No |
| 17/11/2000 | Order no. 1100/2000 | Technical and scientific criteria for changing dispensing status; Rx to OTC switch benefit / risk assessment; exclusion of substances with safety concerns from fixed dose combinations; revoking marketing authorizations | Community Pharmacy | Forms of dispensing medicines | No |
| 25/05/2001 | Dispatch no. 12624/2001 | Electronic prescription model, within the scope of the NHS | Prescription | Electronic Prescription | No |
| 29/05/2001 | Law no. 12/2001 | Emergency contraception | Community Pharmacy | Forms of dispensing medicines | No |
| 30/05/2001 | Order no. 543/2001 | Reimbursement levels for pharmacotherapeutic groups and subgroups, reimbursement increased for neuroleptic and antidepressant drugs when prescribed by general practitioner also | Reimbursement | Reimbursement List | No |
| 07/06/2001 | Order no. 577/2001 | Public price of generic drugs should be at least 35% lower than the retail price of the reference drug, with the same dosage and in the same pharmaceutical form | Generic policies | Generic Price link | No |
| 03/08/2001 | Law no. 84/2001 | Switching from existing pharmaceutical specialties to generic drugs | Generic policies | Generics Regulation | No |
| 03/10/2001 | Protocol | Protocol between the Ministry of Health and the Pharmaceutical Industry, on 03 October 2001 | Others | Claw-back (and other measures applied in the case of excess spending in pharmaceutical budget) | Yes |
| 18/10/2001 | Dispatch no. 23059/2001 | Protocol between the government and the Pharmaceutical Industry, defining a cap for expenditure and payback for the years 2001, 2002 and 2003 | Others | Claw-back (and other measures applied in the case of excess spending in pharmaceutical budget) | Yes |
| 08/11/2001 | Dispatch no. 24475/2001 | Extension of the reimbursement of the presentations of 56 and 60 of PPIs | Reimbursement | Reimbursement List | No |
| 14/11/2001 | Order no. 1278/2001 | Adequacy of packaging size, therapeutic indication dosage and the duration of treatment for reimbursed medicines | Reimbursement | Reimbursement List | No |
| 14/11/2001 | Order no. 1279/2001 | Definition of price update percentages | Pricing | Price Review | No |
| 26/02/2002 | Dispatch no. 5853/2002 | Preservation of documents relating to the prescription of medicines to allow investigation | Prescription | Prescription forms and information systems | No |
| 07/03/2002 | Dispatch no. 7145/2002 | Incentive for prescription by INN with sharing of savings among hospitals and health centers within the NHS | Generic policies | Physicians' incentives | No |
| 25/03/2002 | Resolution no. 728/2002 | Essentially similar medicine concept, reference medicine concept, generic medicine concept in Portugal | Generic policies | Generics Regulation | No |
| 22/10/2002 | Order no. 1379/2002 | Rules and conditions for opening of pharmacies (capitation, distance) | Community Pharmacy | Ownership, licensing, establishment and operation | No |
| 02/12/2002 | Decree-Law no. 270/2002 | Introduction of reference prices in the reimbursement of medicines (highest price generic medicine); 25% increase over the reference price in the special regime | Reimbursement | Reference Price System (RPS) | No |
| 02/12/2002 | Decree-Law no. 271/2002 | Prescription by INN of drugs containing active substances for which there are authorized generic drugs; brand name of the drug or the name of the MA holder being admitted after that indication; medical doctors authorize or refuse the dispensation of generic medication | Generic policies | International Non-proprietary Name Prescribing (INN Prescribing) | No |
| 05/12/2002 | Joint Dispatch no. 865-A/2002 | Reference prices of homogeneous groups; corresponding to the public price of the highest-priced generic of each | Reimbursement | Reference Price System (RPS) | No |
| 05/12/2002 | Order no. 1492-A/2002 | Update of prescription drugs’ prices, generic drugs and non-prescription drugs reimbursed | Pricing | Price Review | No |
| 12/12/2002 | Order no. 1501/2002 | Approval of new single model of uniform medical prescription, for manual or computerized form, enabling it to be renewable, providing adaptation to a fully electronic format | Prescription | Prescription forms and information systems | No |
| 07/01/2003 | Dispatch no. 1389/2003 | End of sharing of savings among hospitals and health centers within the NHS resulting from prescription by INN | Generic policies | Physicians' incentives | No |
| 18/03/2003 | Dispatch no. 7330/2003 | Adaptation of the medical prescription to the electronic format | Prescription | Electronic Prescription | No |
| 16/04/2003 | Dispatch no. 9896/2003 | Access to medicines for the treatment of Alzheimer's | Reimbursement | Reimbursement List | No |
| 22/05/2003 | Dispatch no. 11619/2003 | Update of the therapeutic groups covered by the conditions for prescriptions to patients with chronic renal failure, and kidney transplant recipients | Reimbursement | Reimbursement List | No |
| 30/06/2003 | Dispatch no. 12566-B/2003 | Definition of the reimbursement of glucose test strips, needles, syringes and lancets for diabetics | Reimbursement | Reimbursement List | No |
| 01/09/2003 | Order no. 914/2003 | Public price of a new generic should be at least 35% lower than the reference product and equal to or less than the reference price | Generic policies | Generic Price link | No |
| 11/10/2003 | Decree-Law no. 249/2003 | Stimulate the switch of medicines essentially similar to generics | Generic policies | Generic Substitution | No |
| 11/10/2003 | Dispatch no. 20071-A/2003 | Regulation of the switch of medicines essentially similar to generics (Decree-Law no.249/2003) | Generic policies | Generic Substitution | No |
| 14/10/2003 | Dispatch no. 21212/2003 | Access to medicines for the treatment of Alzheimer's | Reimbursement | Reimbursement List | No |
| 27/09/2003 | Decree-Law no. 234/2003 | Expansion of the generics market, prescription by INN, reimbursement by reference price for homogeneous groups of medicines | Reimbursement | Reference Price System (RPS) | No |
|  |  |  | Generic policies | International Non-proprietary Name Prescribing (INN Prescribing) | No |
| 08/01/2004 | Dispatch no. 2837/2004 | Access to NHS establishments by Medical REPs | Prescription | Pharmaceutical Promotion and interaction with Health Care Professionals (HCPs) | No |
| 06/02/2004 | Decree-Law no. 31/2004 | Extension of the 25% increase for a period of one year over the reference price for the special regime | Reimbursement | Eligibility Scheme(s) | No |
| 18/02/2004 | Order no. 168-B/2004 | Tendering regime for the installation of new pharmacies and their transfer | Community Pharmacy | Ownership, licensing, establishment and operation | No |
| 23/02/2004 | Order no. 172/2004 | Health subsystem (ADSE) is subject to the NHS legal regime | Reimbursement | Eligibility Scheme(s) | No |
| 03/03/2004 | Order no. 236/2004 | Revocation of the ordinance 1100/2000 that established the rules regarding medicines that should be considered as non-prescription | Community Pharmacy | Forms of dispensing medicines | No |
| 10/04/2004 | Decree-Law no. 81/2004 | Increase in the periodicity of definition of reference prices and new homogeneous groups | Reimbursement | Reference Price System (RPS) | No |
| 26/05/2004 | Order no. 561/2004 | Health subsystems ((ADMG and SAD) are subject to SNS legal regime | Reimbursement | Eligibility Scheme(s) | No |
| 02/07/2004 | Dispatch no. 14916/2004 | Update of the therapeutic groups covered by the conditions for prescriptions to patients with chronic renal failure, and kidney transplant recipients | Reimbursement | Reimbursement List | No |
| 21/12/2004 | Order no. 1471/2004 | Definition of the size of the packaging of medicines that can be reimbursed | Reimbursement | Reimbursement List | No |
| 21/12/2004 | Order no. 1474/2004 | Pharmacotherapeutic groups and subgroups that integrate the different reimbursement levels | Reimbursement | Reimbursement List | No |
| 24/01/2005 | Dispatch no. 5080/2005 | Dematerialization of the medical prescription | Prescription | Electronic Prescription | No |
| 26/01/2005 | Decree-Law no. 23/2005 | Extension of the 25% increase over the reference price for users of the special regime for another year | Reimbursement | Eligibility Scheme(s) | No |
| 27/01/2005 | Protocol | Protocol between the Ministry of Health and the Pharmaceutical Industry | Others | Claw-back (and other measures applied in the case of excess spending in pharmaceutical budget) | Yes |
| 17/02/2005 | Resolution no. 292/2005 | Replacement of books and manual records of psychotropic and narcotic drugs with computer records | Prescription | Electronic Prescription | No |
| 05/04/2005 | Order no. 393/2005 | Fixed combinations of antiasthmatics and or bronchodilators, and NSAIDs, move from level C of reimbursement to level B | Reimbursement | Reimbursement List | No |
| 21/06/2005 | Law no. 38/2005 | Authorization for the Government to legislate on distribution outside pharmacies of OTCs | Community Pharmacy | Forms of dispensing medicines | No |
| 27/07/2005 | Order no. 618-A/2005 | Reduction of the sale price of medicines by 6% (new drugs lower ex-factory price by 3% and lower the margins of wholesalers and pharmacies) | Pricing | Price Cut | Yes |
| 11/08/2005 | Decree-Law no. 129/2005 | 5% reduction in the maximum level of reimbursement; eliminating the 10% increase in generics’ reimbursement | Reimbursement | Reimbursement Rate | Yes |
| 16/08/2005 | Decree-Law no. 134/2005 | Regulation of the sale of OTCs outside pharmacies | Community Pharmacy | Forms of dispensing medicines | No |
| 01/09/2005 | Dispatch no. 19650-A/2005 | 100% reimbursement of medicines considered essential in terms of life support | Reimbursement | Eligibility Scheme(s) | No |
| 14/09/2005 | Order no. 826/2005 | Retail prices for all drugs already approved are reduced by 6% | Pricing | Price Cut | Yes |
| 14/09/2005 | Order no. 827/2005 | Definition of the conditions for the sale of non-prescription drugs (OTCs) | Community Pharmacy | Forms of dispensing medicines | No |
| 07/12/2005 | Resolution no. 1706/2005 | Prior registration of points of sale for OTCs | Community Pharmacy | Forms of dispensing medicines | No |
| 03/01/2006 | Decree-Law no. 6/2006 | Extension of the 25% increase on the reference price for the special regime | Reimbursement | Eligibility Scheme(s) | No |
| 27/01/2006 | Order no. 91/2006 | Compulsory presentation of the declaration and the supporting document by pensioners who intend to benefit from the special medication reimbursement regime | Reimbursement | Eligibility Scheme(s) | No |
| 10/02/2006 | Protocol no. 7/2006 | Protocol between the Ministry of Health and the Pharmaceutical Industry. | Others | Claw-back (and other measures applied in the case of excess spending in pharmaceutical budget) | Yes |
| 10/03/2006 | Order no. 257/2006 | Regulation of the National Council for the Advertising of Medicines | Prescription | Pharmaceutical Promotion and interaction with Health Care Professionals (HCPs) | No |
| 22/03/2006 | Dispatch no. 8161/2006 | Humalog reimbursement | Reimbursement | Reimbursement List | No |
| 28/04/2006 | Dispatch no. 11118/2006 | Switching from branded medicines to generics | Generic policies | Generics Regulation | No |
| 17/05/2006 | Dispatch no. 12188/2006 | Deadline to benefit from the special reimbursement regime | Reimbursement | Eligibility Scheme(s) | No |
| 25/05/2006 | Dispatch no. 12589/2006 | Definition of the procedures for the special reimbursement regime | Reimbursement | Eligibility Scheme(s) | No |
| 04/07/2006 | Decree-Law no. 127/2006 | Extension of the 25% increase over the reference price for the special regime for another year | Reimbursement | Eligibility Scheme(s) | No |
| 24/07/2006 | Order no. 728/2006 | Adaptation of the special regime for the reimbursement of medicines to public administration employees and agents (ADSE) | Reimbursement | Eligibility Scheme(s) | No |
| 30/11/2006 | Dispatch no. 25909/2006 | Update of the therapeutic groups covered by the conditions for prescriptions to patients with chronic renal failure, and kidney transplant recipients | Reimbursement | Reimbursement List | No |
| 06/12/2006 | Decree-Law no. 235/2006 | Opening of retail pharmacies in NHS hospitals | Community Pharmacy | Ownership, licensing, establishment and operation | No |
| 29/12/2006 | Decree-Law no. 242-A/2006 | Extension of the increase, but reduction to 20%, on the reference price for the special regime | Reimbursement | Eligibility Scheme(s) | Yes |
| 29/12/2006 | Law no. 53-A/2006 | Retail price for approved reimbursed drugs is reduced by 6%; margin of 6.87% and 18.25% for wholesalers and pharmacies; reduces the reimbursement rates B, C and D (69%, 37% and 15%) | Pricing | Price Cut | Yes |
|  |  |  | Reimbursement | Reimbursement Rate | Yes |
|  |  |  | Community Pharmacy | Distribution Remuneration | Yes |
| 05/01/2007 | Order no. 30-B/2007 | 6% price reduction in retail price for all reimbursed drugs approved by December 31, 2006; margins are changed to 6.87% for the wholesaler and 18.25% for the pharmacy | Pricing | Price Cut | Yes |
|  |  |  | Community Pharmacy | Distribution Remuneration | Yes |
| 08/03/2007 | Decree-Law no. 53/2007 | Regulation of the working hours of retail pharmacies | Community Pharmacy | Ownership, licensing, establishment and operation | No |
| 14/03/2007 | Decree-Law no. 65/2007 | Reference countries definition (now includes Greece); annual reviews | Pricing | External Price Referencing (EPR) | Yes |
| 19/03/2007 | Order no. 300-A/2007 | Establishment of the rules for the setting of new drug prices, annual review of prices, with a reduction or increase of up to 2.5% to the practiced retail price, or if there is no identical or similar pharmaceutical specialty in the reference countries, remains unchanged; gradual annual price reduction of up to 10% (2007 and 2008); price reduction for generics: 5% (share 50% - 60%), 4% (share 60%-70%), 3% (share> 70%) | Pricing | Price Cut | Yes |
| 27/04/2007 | Dispatch no. 10053/2007 | Update of the therapeutic groups covered by the conditions for prescriptions to patients with chronic renal failure, and kidney transplant recipients | Reimbursement | Reimbursement List | No |
| 12/06/2007 | Law no. 20/2007 | Authorization for the Government to legislate on the property of pharmacies and to adapt the general regime of administrative offenses committed in the exercising of pharmaceutical activity | Community Pharmacy | Ownership, licensing, establishment and operation | No |
| 19/06/2007 | Decree-Law no. 238/2007 | To allow OTCs to be sold outside pharmacies but without reimbursement; switching from Rx-only to OTCs, when it already exists as OTC and with possible exclusion from reimbursement | Community Pharmacy | Forms of dispensing medicines | No |
|  |  |  | Reimbursement | De-listing (Delisting) | No |
| 31/08/2007 | Decree-Law no. 307/2007 | Legal framework for retail pharmacies (individuals or commercial companies); mandatory shareholder identification; duties of the Technical Director; two pharmacists per pharmacy; rules for transfers | Community Pharmacy | Ownership, licensing, establishment and operation | No |
| 02/11/2007 | Order no. 1427/2007 | Regulation of the conditions and requirements for home-delivery and over-the-Internet medicines | Community Pharmacy | Forms of dispensing medicines | No |
| 02/11/2007 | Order no. 1430/2007 | Licensing and granting procedures for new pharmacies and those resulting from the transformation of permanent pharmaceutical posts, as well as the transfer of the location of pharmacies | Community Pharmacy | Ownership, licensing, establishment and operation | No |
| 28/12/2007 | Decree-Law no. 392-B/2007 | Extension of the 20% increase over the reference price for the special regime | Reimbursement | Eligibility Scheme(s) | No |
| 31/12/2007 | Law no. 67-A/2007 | Revision of the system for setting the margins for the sale of reimbursed medicines at wholesalers and in pharmacies | Community Pharmacy | Distribution Remuneration | No |
| 07/02/2008 | Determination no. 44/CD/2008 | Essential information to include in advertising pieces, dimensions, number of samples to distribute | Prescription | Pharmaceutical Promotion and interaction with Health Care Professionals (HCPs) | No |
| 11/03/2008 | Dispatch no. 10279/2008 | Reimbursement at level A (95%) of opioid drugs, when prescribed for the treatment of moderate to severe cancer pain | Reimbursement | Reimbursement Rate | No |
| 11/03/2008 | Dispatch no. 10280/2008 | Reimbursement at level A (95%) of opioid drugs, when prescribed for the treatment of chronic non-cancer pain, moderate to severe and referred to in Pain Units | Reimbursement | Reimbursement Rate | No |
| 27/06/2008 | Decree-Law no. 110/2008 | Extension of the 20% increase on the reference price for the special regime | Reimbursement | Eligibility Scheme(s) | No |
| 08/09/2008 | Order no. 1016-A/2008 | 30% reduction of the maximum retail prices for the sale of generic medicines approved until 31 March 2008 | Generic policies | Price Cut | Yes |
| 18/12/2008 | Decree-Law no. 247/2008 | Extension of the 20% increase on the reference price for the special regime | Reimbursement | Eligibility Scheme(s) | No |
| 10/02/2009 | Order no. 157/2009 | Approval of the Regulation of the National Council for the Advertising of Medicines | Prescription | Pharmaceutical Promotion and interaction with Health Care Professionals (HCPs) | No |
| 14/05/2009 | Dispatch no. 12220/2009 | Update on reimbursement - opioid drugs, treatment of chronic non-cancer pain | Reimbursement | Reimbursement List | No |
| 14/05/2009 | Dispatch no. 12221/2009 | Update on reimbursement - opioid drugs, treatment of chronic cancer pain | Reimbursement | Reimbursement List | No |
| 29/05/2009 | Decree-Law no. 129/2009 | Reimbursement in the special regimen is increased by 5% in level A and 15% in B, C and D; for generics it is 100% for all levels | Generic policies | Patients' incentives | No |
|  |  |  | Reimbursement | Reimbursement Rate | No |
| 12/06/2009 | Order no. 650/2009 | Establishment of the procedures to the special regime for the reimbursement of medicines to ADM beneficiaries | Reimbursement | Eligibility Scheme(s) | No |
| 19/06/2009 | Order no. 668/2009 | Maintaining the exception in terms of effects regarding reference prices for generics | Generic policies | Generic Price link | No |
| 01/07/2009 | Order no. 697/2009 | Regulation of the dispensing of medications in unit dose in the outpatient sector | Community Pharmacy | Forms of dispensing medicines | No |
| 07/08/2009 | Decree-Law no. 182/2009 | Simplification of the procedure leading to parallel import of medicines | Pricing | Parallel Trade | No |
| 15/09/2009 | Order no. 1047/2009 | Maintaining the exception in terms of effects regarding reference prices for generics | Generic policies | Generic Price link | No |
| 16/09/2009 | Decree-Law no. 241/2009 | Establishment of the regime for the installation, opening and operation of retail pharmacies for dispensing medicines to the public in NHS hospitals | Community Pharmacy | Ownership, licensing, establishment and operation | No |
| 12/10/2009 | Regulatory Decree no. 28/2009 | Definition of the terms of the adaptation of the prescription model for narcotic drugs and psychotropics to the electronic form | Prescription | Electronic Prescription | No |
| 15/10/2009 | Order no. 1263/2009 | Fixed combinations of antiasthmatics and or bronchodilators (5.1) move from level C to level B | Reimbursement | Reimbursement List | No |
| 31/12/2009 | Order no. 1460-D/2009 | Maintaining the exception in terms of effects regarding reference prices for generics | Generic policies | Generic Price link | No |
| 08/01/2010 | Order no. 16/2010 | Maintaining the exception in terms of effects regarding reference prices for generics | Generic policies | Generic Price link | No |
| 15/01/2010 | Decree-Law no. 6/2010 | Extension of the 20% increase on the reference price for the special regime | Reimbursement | Eligibility Scheme(s) | No |
| 11/03/2010 | Order no. 154-A/2010 | Exceptional postponement, in 2010, of the annual review of prices | Pricing | External Price Referencing (EPR) | No |
| 07/05/2010 | Law no. 6/2010 | Inclusion in level A of reimbursement keratolytic and antipsoriatic drugs for patients with psoriasis | Reimbursement | Reimbursement List | No |
| 13/05/2010 | Decree-Law no. 48-A/2010 | 100% reimbursement for the special drug regime to the 5 lowest retail prices in the respective homogeneous; shortening of the decision-making period for the request for reimbursement of generic medicines; where there is already a homogeneous group, the retail price of new generics must be 5% lower than the maximum retail price of the lowest priced generic; starting from the 5th generic drug, inclusive, 5% price reduction in the generic whose request for reimbursement is immediately beforehand | Generic policies | Patients' incentives | No |
|  |  |  | Generic policies | Generic Price link | Yes |
| 25/05/2010 | Order no. 283/2010 | Maintaining the exception in terms of effects regarding reference prices for generics | Generic policies | Generic Price link | No |
| 11/06/2010 | Order no. 312-A/2010 | Changes in the methodology for defining and reviewing prices of generics and non-generics | Pricing | Price Review | Yes |
|  |  |  | Generic policies | Generic Price link | Yes |
| 16/06/2010 | Order no. 337-A/2010 | Clarification of the annual review of non-generic drug prices | Pricing | Price Review | Yes |
| 30/06/2010 | Order no. 455-A/2010 | Regulation of the dispensing of medications in unit dose in the outpatient sector | Community Pharmacy | Forms of dispensing medicines | No |
| 29/06/2010 | Law no. 12-A/2010 | Value-added tax (VAT) on medicines increased to 6% | Others | Changes in the value-added tax (VAT) on medicines | Yes |
| 22/07/2010 | Dispatch no. 12455/2010 | Pathological situations that benefit from full reimbursement in the administration of growth hormone | Reimbursement | Reimbursement List | No |
| 01/02/2010 | Dispatch no. 2623/201 | Pathological situations that benefit from full reimbursement of growth hormone | Reimbursement | Reimbursement List | No |
| 17/09/2010 | Order no. 924-A/2010 | Transfer of NSAIDs and PPIs to level C of reimbursement; ends the increase in the share of antidepressants in the case of some pathologies; maintains fixed dose combinations of antiasthmatics and / or bronchodilators in level B | Reimbursement | Reimbursement List | Yes |
| 29/09/2010 | Order no. 994-A/2010 | Subgroups 10.1 - Antihistamines and 10.1.1 - H1 sedative antihistamines are added to level C of reimbursement | Reimbursement | Reimbursement List | No |
| 01/10/2010 | Decree-Law no. 106-A/2010 | Reference price becomes the average of the five cheapest drugs on the market that integrate each homogeneous group and not the generic with the highest retail price; the reimbursement for level A in the regular regime is reduced from 95% to 90%; ; the reimbursement for level A in the special regime is reduced from 95% to 90%, there is no longer 100%; reimbursement just for prescriptions by electronic systems | Reimbursement | Reference Price System (RPS) | Yes |
| 07/10/2010 | Order no. 1041-A/2010 | 6% reduction of maximum authorized retail prices for reimbursed human medicines, without changing margins | Pricing | Price Cut | Yes |
| 14/10/2010 | Order no. 1056-B/2010 | Subgroup 10.1.2 - non-sedative H 1 antihistamines are also added to level C | Reimbursement | Reimbursement List | No |
| 16/08/2010 | Order no. 707/2010 | Introduction of vaccines and immunoglobulins to level C of reimbursement | Reimbursement | Reimbursement List | No |
| 27/12/2010 | Resolution of the Council of Ministers no. 101-A/2010 | Expenditure control measures – stability and growth program | All |  | Yes |
| 28/12/2010 | Order no. 1319/2010 | Definition of the conditions for the special drug reimbursement regime | Reimbursement | Eligibility Scheme(s) | No |
| 02/01/2011 | Order no. 3/2012 | Exceptional drug price review for reasons of public interest or at the initiative of the MAH | Pricing | Price Review | No |
| 10/01/2011 | Decree-Law no. 7/2011 | Authorization of pharmacies opening twenty-four hours a day, seven days a week, in conjunction with the shift regime, | Community Pharmacy | Ownership, licensing, establishment and operation | No |
| 11/01/2011 | Order no. 31-A/2011 | Definition of the minimum limit of the working week, daily working period of the pharmacy, shift schedules, charges by shift pharmacies | Community Pharmacy | Ownership, licensing, establishment and operation | No |
| 09/02/2011 | Dispatch no. 2826/2011 | Criteria, deadlines and procedures for the exceptional review of drug prices | Pricing | Price Review | No |
| 22/03/2011 | Order no. 112-B/2011 | Exceptional postponement in 2011 of the annual price review, as there was already a 6% deduction to be applied on the maximum authorized retail prices | Pricing | External Price Referencing (EPR) | No |
| 13/05/2011 | Order no. 193/2011 | Regulation of the procedure for the reimbursement of medicines dispensed to beneficiaries of the NHS who are not covered by any subsystem or who benefit from a complementary contribution | Reimbursement | Eligibility Scheme(s) | No |
| 18/05/2011 | Order no. 198/2011 | Rules for electronic prescription with paper printing, until there is total dematerialization | Prescription | Electronic Prescription | No |
| 16/06/2011 | Law no. 25/2011 | Restoration of obligatory indication of retail price on labeling of medicines | Others | Price Labelling | No |
| 17/06/2011 | Dispatch no. 8680/2011 | Update of the therapeutic groups covered by the conditions for prescriptions to patients with chronic renal failure, and kidney transplant recipients | Reimbursement | Reimbursement List | No |
| 15/07/2011 | Dispatch no. 9187/2011 | Definition of the exceptions to the electronic prescription of medicines | Prescription | Electronic Prescription | No |
| 15/09/2011 | Order no. 267-A/2011 | Definition of the conditions for the inclusion of new drugs in the respective special reimbursement regime, whether they are drugs used in the treatment of certain pathologies or by special groups of users | Reimbursement | Reimbursement List | No |
| 02/11/2011 | Dispatch no. 15096/2011 | Updating vignettes for the electronic prescription exception regime | Prescription | Prescription forms and information systems | No |
| 03/11/2011 | Order no. 289-A/2011 | Continuation in force until 1 December 2011 of the fixed combinations of antiasthmatics and or bronchodilators (5.1) in level B of reimbursement | Reimbursement | Reimbursement List | No |
| 29/11/2011 | Decree-Law no. 112/2011 | Change to reference countries (Spain, Italy and Slovenia); generic drugs price at least 50% lower than the retail price of the reference product (or 25% if retail price is less than € 10 in all presentations); change in maximum margins for reimbursed and non-reimbursed drugs; discounts are allowed throughout the medicine circuit, from the manufacturer to the retailer | Pricing | External Price Referencing (EPR) | Yes |
|  |  |  | Generic policies | Generic Price link | Yes |
|  |  |  | Community Pharmacy | Distribution Remuneration | No |
|  |  |  | Community Pharmacy | Discounts, rebates, loyalty schemes | No |
| 30/11/2011 | Order no. 300/2011 | Continuation of the fixed combinations of antiasthmatics and or bronchodilators (5.1) in level B of reimbursement | Reimbursement | Reimbursement List | No |
| 12/12/2011 | Law no. 62/2011 | Arbitration required for generics entry, end of patent linkage | Generic policies | Breakdown artificial barriers for generics market access (e.g. patent linkage) | No |
| 02/01/2012 | Order no. 4/2012 | Price changes made at the initiative of MAH; annual price reviews of non-generics with the exception of presentations with PVP in force less than or equal to € 5.00; annual review of generic prices remains at 50% and 25%, with the exception of presentations with retail prices less than or equal to € 3.25 | Pricing | Price Review | No |
| 02/01/2012 | Order no. 3/2012 | Exceptional drug price review for reasons of public interest or at the initiative of the MAH | Pricing | Price Review | No |
| 02/01/2012 | Order no. 4/2012 | Establishment of the rules for the annual price review | Pricing | External Price Referencing (EPR) | No |
|  |  |  | Generic policies | Generic Price link | Yes |
| 13/02/2012 | Order no. 46/2012 | Special vignette safety mechanisms and measures that guarantee the integrity of the system associated with manual prescription | Prescription | Prescription forms and information systems | No |
| 08/03/2012 | Law no. 11/2012 | INN prescription; pharmacies must have 3 of the 5 cheapest generics available | Generic policies | International Non-proprietary Name Prescribing (INN Prescribing) | No |
| 11/05/2012 | Order no. 137-A/2012 | Prescription by INN | Generic policies | International Non-proprietary Name Prescribing (INN Prescribing) | No |
| 14/05/2012 | Protocol | Reduction of expenditure to the amount of 300 million euros, compared to the values ​​verified in 2011, with a decrease in public expenditure in the hospital market of 170 million euros and in the ambulatory market of 130 million euros | Others | Claw-back (and other measures applied in the case of excess spending in pharmaceutical budget) | Yes |
| 12/07/2012 | Decree-Law no. 152/2012 | Transference of responsibility for drug pricing to the Ministry of Health; Retail price of the reference product is determined by the average of that product in the two years immediately preceding the price request for the first generic medicine | Pricing | Price Set | No |
|  |  |  | Generic policies | Generic Price link | No |
| 01/08/2012 | Decree-Law no. 172/2012 | Regulation of working hours of retail pharmacies | Community Pharmacy | Ownership, licensing, establishment and operation | No |
| 12/09/2012 | Order no. 277/2012 | Authorization of pharmacies opening twenty-four hours a day, seven days a week, in conjunction with the shift regime, | Community Pharmacy | Ownership, licensing, establishment and operation | No |
| 04/10/2012 | Dispatch no. 13381/2012 | Mandatory use of new vignette models | Prescription | Prescription forms and information systems | No |
| 18/10/2012 | Dispatch no. 13901/2012 | Communication of data on the prescription of medicines dispensed in community pharmacies that have been prescribed in the context of private medicine and reimbursed by the NHS to the respective prescribers | Prescription | Prescription forms and information systems | No |
| 25/10/2012 | Order no. 340/2012 | Creation of the Pharmacy and Therapeutic Commissions for each Regional Health Administration (CFT-ARS) | Prescription | Prescription forms and information systems | No |
| 30/10/2012 | Order no. 352/2012 | Regulation of the licensing and granting procedure for new pharmacies, as well as the transfer of the location of pharmacies and the endorsement of the license | Community Pharmacy | Ownership, licensing, establishment and operation | No |
| 30/11/2012 | Dispatch no. 15700/2012 | Approval of the prescription form models | Prescription | Prescription forms and information systems | No |
| 14/12/2012 | Order no. 411-A/2012 | Suspension of the application of the deadlines established for the purpose of the annual review of drug prices for 2013 | Pricing | External Price Referencing (EPR) | No |
| 14/12/2012 | Order no. 407/2012 | Creation of the pharmaceutical industry special contributions management fund for NHS stabilization | Others | Claw-back (and other measures applied in the case of excess spending in pharmaceutical budget) | Yes |
| 11/01/2013 | Order no. 14/2013 | Opening hours, shift schedules, maximum amount to be charged by shift pharmacies | Community Pharmacy | Ownership, licensing, establishment and operation | No |
| 08/02/2013 | Law no. 16/2013 | Review of the legal regime for retail pharmacies | Community Pharmacy | Ownership, licensing, establishment and operation | No |
| 14/02/2013 | Decree-Law no. 20/2013 | Transparency in the relationship between the Pharmaceutical industry and health professionals; communication on the INFARMED website of promotional activities | Prescription | Pharmaceutical Promotion and interaction with Health Care Professionals (HCPs) | No |
| 27/02/2013 | Decree-Law no. 34/2013 | Approval of the regime for the setting of the price of medicines: for hospital segment the reference is the lowest price in European countries in comparison; for retail market the comparative GDP per capita in terms of purchasing power parity or a lower price level is introduced as a criterion for comparators | Pricing | External Price Referencing (EPR) | Yes |
| 28/02/2013 | Order no. 91/2013 | Reference countries to consider in 2013: Slovakia, Spain and France | Pricing | External Price Referencing (EPR) | Yes |
| 04/03/2013 | Dispatch no. 4005/2013 | Reimbursement of medicines dispensed to beneficiaries of health subsystems becomes the responsibility of the NHS | Reimbursement | Eligibility Scheme(s) | No |
| 13/03/2013 | Dispatch no. 4138/2013 | Definition of what is meant by objects of negligible value and relevant to the practice of medicine or pharmacy | Prescription | Pharmaceutical Promotion and interaction with Health Care Professionals (HCPs) | No |
| 28/03/2013 | Order no. 135-B/2013 | Establishment of the reference countries and the deadlines for the annual review of drug prices for 2013 | Pricing | External Price Referencing (EPR) | Yes |
| 24/06/2013 | Dispatch no. 8213-B/2013 | Rules for access by REPs to NHS services and establishments | Prescription | Pharmaceutical Promotion and interaction with Health Care Professionals (HCPs) | No |
| 26/07/2013 | Decree-Law no. 103/2013 | Implementation of a minimum 5% market share of generic drugs in a homogeneous group to identify the cheapest generics to be used as a reference for the retail price of new medicines. | Reimbursement | Reference Price System (RPS) | No |
| 01/07/2013 | Dispatch no. 8990-C/2013 | Medical prescription templates and vignette placement | Prescription | Prescription forms and information systems | No |
| 09/07/2013 | Order no. 224-A/2013 | There is no longer a requirement for physical segregation between different types of prescription | Prescription | Prescription forms and information systems | No |
| 30/08/2013 | Dispatch no. 11254/2013 | Change the medical prescription form models | Prescription | Prescription forms and information systems | No |
| 05/09/2013 | Decree-Law no. 128/2013 | Transparency in the relationship between the Pharmaceutical industry and health professionals; communication on the INFARMED website of promotional activities | Prescription | Pharmaceutical Promotion and interaction with Health Care Professionals (HCPs) | No |
| 24/09/2013 | Protocol | Protocol for 2013 | Others | Claw-back (and other measures applied in the case of excess spending in pharmaceutical budget) | Yes |
| 26/09/2013 | Resolution no. 2092/2013 | Mandatory reporting of intra-Community exported medicines | Pricing | Parallel Trade | No |
| 26/09/2013 | Resolution no. 2100/2013 | Mandatory reporting of intra-Community exported medicines | Pricing | Parallel Trade | No |
| 15/11/2013 | Order no. 335-A/2013 | Reference countries to be considered in 2014: Slovenia, Spain and France | Pricing | External Price Referencing (EPR) | Yes |
| 13/12/2013 | Order no. 367/2013 | Suspension of annual generics price review | Generic policies | Generic Price link | No |
| 31/01/2014 | Order no. 24/2014 | Regulation of the procedure for the reimbursement of medicines dispensed to beneficiaries of the NHS who are not covered by any subsystem or who benefit from a complementary contribution | Reimbursement | Eligibility Scheme(s) | No |
| 05/02/2014 | Decree-Law no. 19/2014 | Rock bottom for generics price defined at maximum of 20% of the retail price of the reference medicine; new pharmacy and wholesaler margins with the introduction of fixed and variable margins, regressive according to the retail price | Generic policies | Generic Price link | No |
|  |  |  | Community Pharmacy | Distribution Remuneration | No |
| 13/02/2014 | Dispatch no. 2977/2014 | Approval of the pharmacotherapeutic classification of drugs | Reimbursement | Reimbursement List | No |
| 21/02/2014 | Order no. 45/2014 | Approval of the pharmacotherapeutic classification of drugs | Reimbursement | Reimbursement List | No |
| 24/02/2014 | Statement of Rectification no. 11-A/2014 | Rectification of the pharmacotherapeutic groups and subgroups that integrate the different levels of co-participation | Reimbursement | Reimbursement List | No |
| 02/04/2014 | Dispatch no. 4742/2014 | Approval of the pharmacotherapeutic classification of drugs | Reimbursement | Reimbursement List | No |
| 03/04/2014 | Order no. 78/2014 | Approval of the pharmacotherapeutic classification of drugs | Reimbursement | Reimbursement List | No |
| 24/06/2014 | Protocol | Agreement for 2014 | Others | Claw-back (and other measures applied in the case of excess spending in pharmaceutical budget) | Yes |
| 10/07/2014 | Decree-Law no. 109/2014 | Legal regime for retail pharmacies | Community Pharmacy | Ownership, licensing, establishment and operation | No |
| 25/08/2014 | Law no. 51/2014 | Fines | Community Pharmacy | Forms of dispensing medicines | No |
| 29/08/2014 | Dispatch no. 11042-F/2014 | Approval of the prescription form model recognized in any Member State of the European Union | Prescription | Prescription forms and information systems | No |
| 12/11/2014 | Order no. 231-A/2014 | Reference countries to be considered in 2015 | Pricing | External Price Referencing (EPR) | Yes |
| 21/11/2014 | Protocol | Agreement for 2015 | Others | Claw-back (and other measures applied in the case of excess spending in pharmaceutical budget) | Yes |
| 19/12/2014 | Dispatch no. 57/2014 | Update on reimbursement - opioid drugs, treatment of chronic cancer pain | Reimbursement | Reimbursement List | No |
| 23/12/2014 | Dispatch no. 251/2009 | Update on reimbursement - opioid drugs, treatment of chronic non-cancer pain | Reimbursement | Reimbursement List | No |
| 31/12/2014 | Law no. 82-B/2014 | Government budget (Pharmaceutical Industry Contribution) | Others | Claw-back (and other measures applied in the case of excess spending in pharmaceutical budget) | Yes |
| 02/02/2015 | Order no. 18-A/2015 | Payment of additional remuneration to pharmacies for increasing the share of generics | Generic policies | Pharmacist' incentives | No |
| 01/06/2015 | Decree-Law no. 97/2015 | Creation of the National Health Technology Assessment System (SiNATS) | Reimbursement | Health Technology Assessment (HTA) | No |
| 30/06/2015 | Order no. 195-A/2015 | Common administrative procedure for reimbursement and prior assessment of medicines | Reimbursement | Health Technology Assessment (HTA) | No |
| 30/06/2015 | Order no. 195-C/2015 | Rules and procedures for the definition, changing and revising of the prices of prescription medicines and non-prescription medicines that are reimbursed, as well as the respective market margins | Community Pharmacy | Distribution Remuneration | No |
|  |  |  | Generic policies | Generic Price link | No |
|  |  |  | Pricing | External Price Referencing (EPR) | Yes |
| 30/06/2015 | Order no. 195-D/2015 | Approval of the pharmacotherapeutic classification of medicines | Reimbursement | Reimbursement List | No |
| 17/07/2015 | Dispatch no. 7979-P/2015 | Progressive standardization of electronic medical prescription tools | Prescription | Electronic Prescription | No |
| 27/07/2015 | Order no. 223/2015 | Regulation of the procedure for the payment of the State's contribution in the PVP for medicines dispensed to beneficiaries of the NHS who are not covered by any subsystem or who benefit from a complementary contribution | Reimbursement | Eligibility Scheme(s) | No |
| 27/07/2015 | Order no. 224/2015 | Consolidation of the extension and adaptation of the rules for the prescription of medicines, the models of medical prescription and the conditions for dispensing medicines with the dematerialization of the prescription | Prescription | Electronic Prescription | No |
| 31/07/2015 | Dispatch no. 9002/2015 | Approval of the model for the treatment guide for dematerialized prescription | Prescription | Electronic Prescription | No |
| 14/10/2015 | Decree-Law no. 238/2015 | Legal regime of health advertising practices | Prescription | Pharmaceutical Promotion and interaction with Health Care Professionals (HCPs) | No |
| 10/11/2015 | Dispatch no. 12682-A/2015 | Reference countries to be considered in 2016: Spain, France and Slovakia | Pricing | External Price Referencing (EPR) | Yes |
| 04/12/2015 | Order no. 417/2015 | Clarification of the identification and collection mechanisms in cases where responsibility for the charges lies with a financial entity other than the NHS | Prescription | Prescription forms and information systems | No |
